# Supplementary material for: Mutant Kras- and p16-regulated NOX4 activation overcomes metabolic checkpoints in development of pancreatic ductal adenocarcinoma
Source: Nat Commun. 2017 Feb 24;8:14437. doi: 10.1038/ncomms14437 (PMC5333128; doi:10.1038/ncomms14437)
Supplement: Supplementary Information — Supplementary Figures and Supplementary Tables [file ncomms14437-s1.pdf]

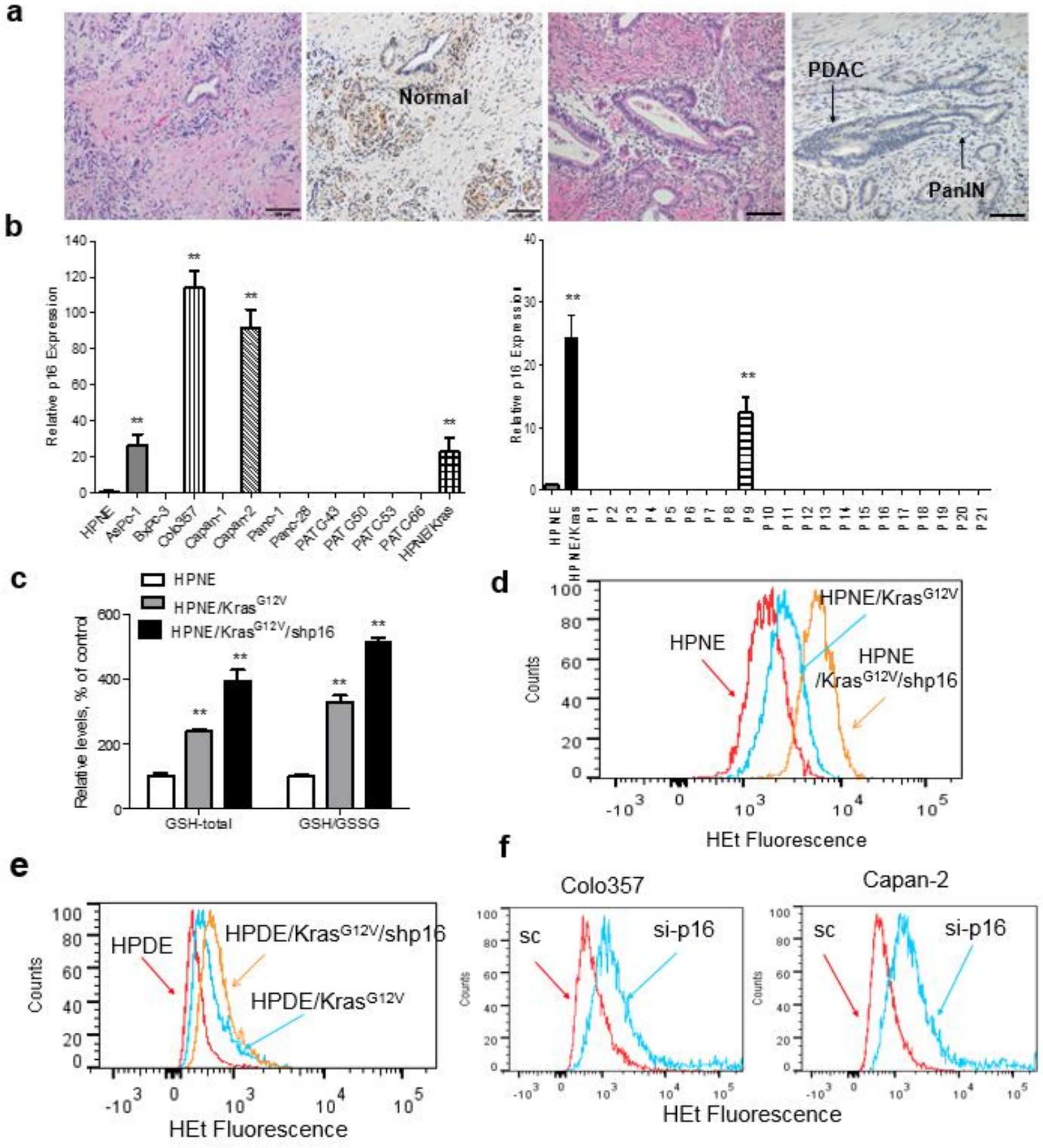

a
Normal
PDAC
PanIN
b
Relative p16 Expression
HPNE
AsPc-1
BxPc-3
Colo357
Capan-1
Capan-2
Panc-1
Panc-28
PATC43
PATC50
PATC53
PATC66
HPNE/Kras
HPNE/Kras
c
HPNE
HPNE/Kras^{G12V}
HPNE/Kras^{G12V}/shp16
Relative levels, % of control
GSH-total
GSH/GSSG
d
HPNE
HPNE/Kras^{G12V}
HPNE /Kras^{G12V}/shp16
Counts
HEt Fluorescence
e
HPDE
HPDE/Kras^{G12V}/shp16
HPDE/Kras^{G12V}
Counts
HEt Fluorescence
f
Colo357
Capan-2
sc
si-p16
Counts
HEt Fluorescence

Supplementary Figure 1. Measurements of p16 expression in PDAC, and the redox status in Kras activated or p16 inactivated HPNE, HPDE and PDAC cells. (a) Representative IHC staining shows the loss of p16 expression in PanIN and PDAC tissues. (Scale bar: 100 μm). (b) The expression of p16 in PDAC cell lines and tissues was analyzed by qPCR assay. (c) The cellular GSH level and GSH/GSSG ratio was compared in these cells. GSH, reduced glutathione; GSSG, oxidized glutathione. (d, e, f) Representative histograms of O_2^- levels in indicated cells as detected by the fluorescent probe Het. Each bar represents the mean ± s.d. (N=3). $^{**}P < 0.01$ for indicated comparison (Student unpaired *t*-test).

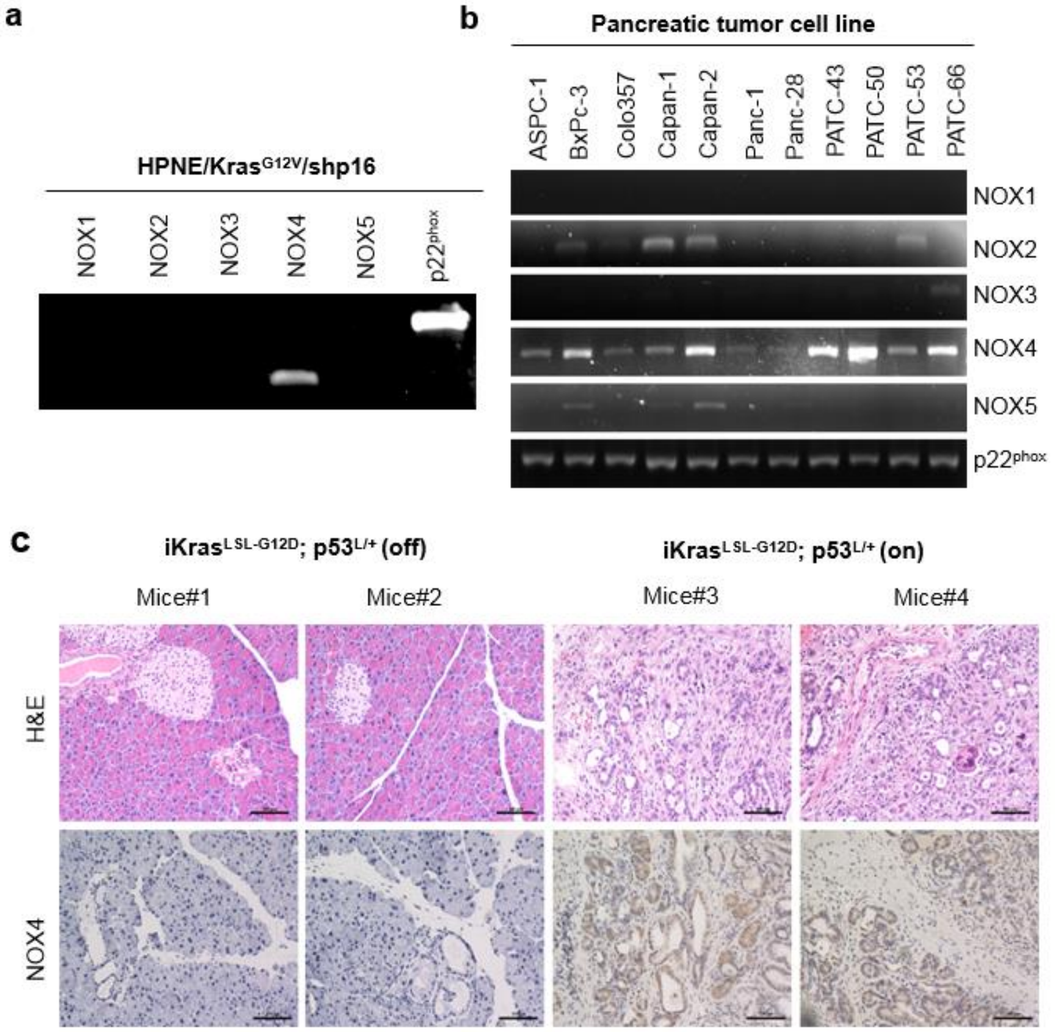

Supplementary Figure 2. NOX4 was specially expressed in PDAC cells, and also increased in transgenetic iKras; p53$^{L/+}$ mice. (a, b) RT-PCR analysis the mRNA expression levels of NOX family members (NOX1, NOX2, NOX3, NOX4, NOX5, p22^{phox}) in HPNE/Kras^{G12V}/shp16 and 11 different PDAC cells. (c) Representative IHC staining with H&E or anti-NOX4 antibody in sections of formalin-fixed PDAC from transgenetic *iKras; p53$^{L/+}$* mice (Scale bar: 100 µm) .

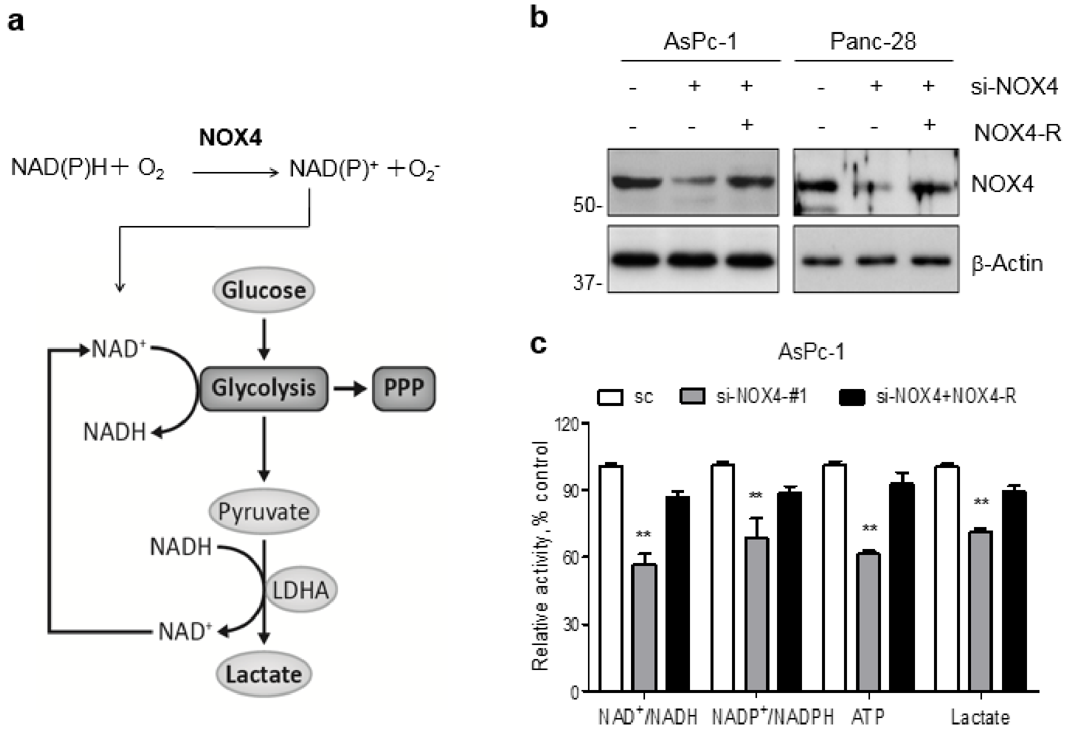

Supplementary Figure 3. Upregulation of NOX4 is required for regeneration of NAD^+ to maintain high glycolytic activity in PDAC cells. (a) Role of NAD^+ in supporting glycolysis. LDHA preferentially converts accumulating pyruvate to lactate, thereby regenerating NAD^+ from NADH to maintain glycolysis. (b) Immunoblotting analysis showed the expression level of NOX4 in AsPc-1 cells after siRNA depletion of NOX4 or co-expressing siRNA resistant NOX4 (NOX4-R). (c) Glycolytic activity (NAD^+/NADH, $NADP^+$/NADPH, ATP and Lactate level) was measured after siRNA depletion of NOX4 or co-expressing NOX4-R in AsPc-1 cells. Each bar represents the mean ± s.d. (n=3). ***P* < 0.01 for indicated comparison (one-way analysis of variance (ANOVA) with the Newman Keul's multiple comparison test).

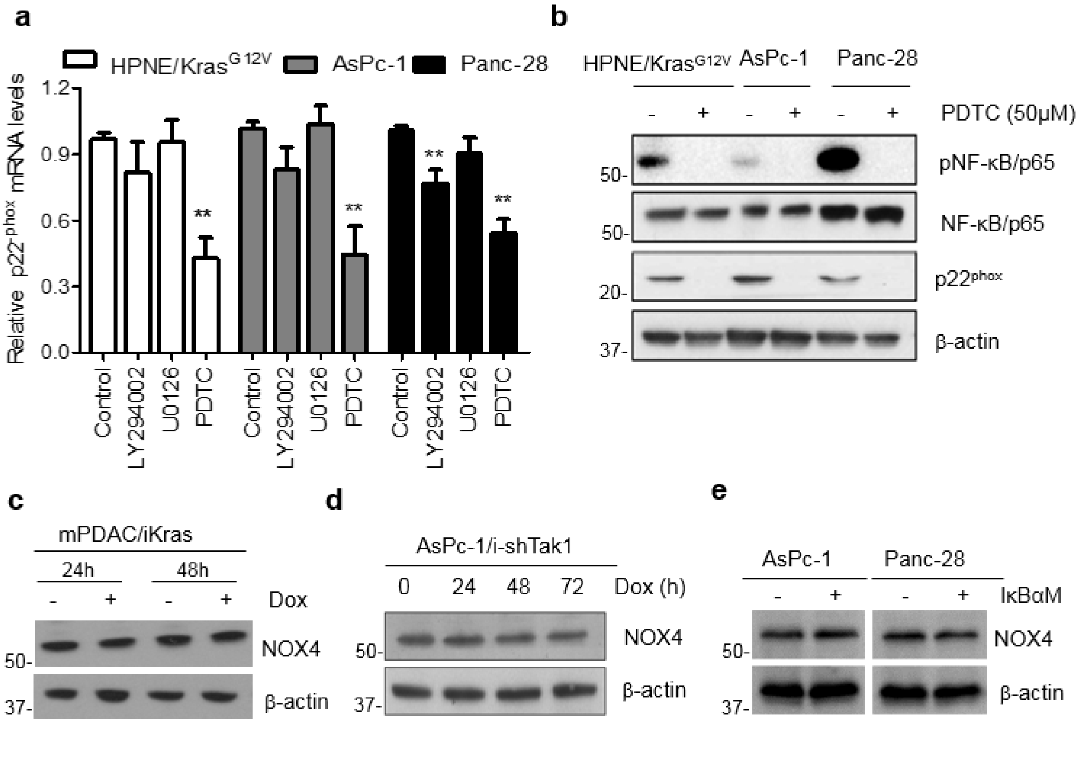

Supplementary Figure 4. Mutant Kras upregulates $p22^{phox}$ expression via the NF-κB pathway. (a) The expression of $p22^{-phox}$ mRNA level was analyzed by qPCR in the indicated cells treated with Akt inhibitor LY294002 (10μM), ERK1/2 inhibitor U0126 (10μM) and NF-κB inhibitor PDTC (20μM) for 48h. (b) The expression of $p22^{-phox}$ protein level was analyzed by Immunoblotting in the indicated cells treated with NF-κB inhibitor PDTC (20μM) for 48h. (c) The expression NOX4 was analyzed by immunoblotting in mPDAC/iKras cells with doxycycline-inducible mutant Kras. (d, e) The expression of NOX4 was analyzed by immunoblotting in indicated cells. β-actin was used as the internal loading control. Each bar represents the mean ± s.d. (n=3). $^{**}P < 0.01$ for indicated comparison (Student unpaired *t*-test).

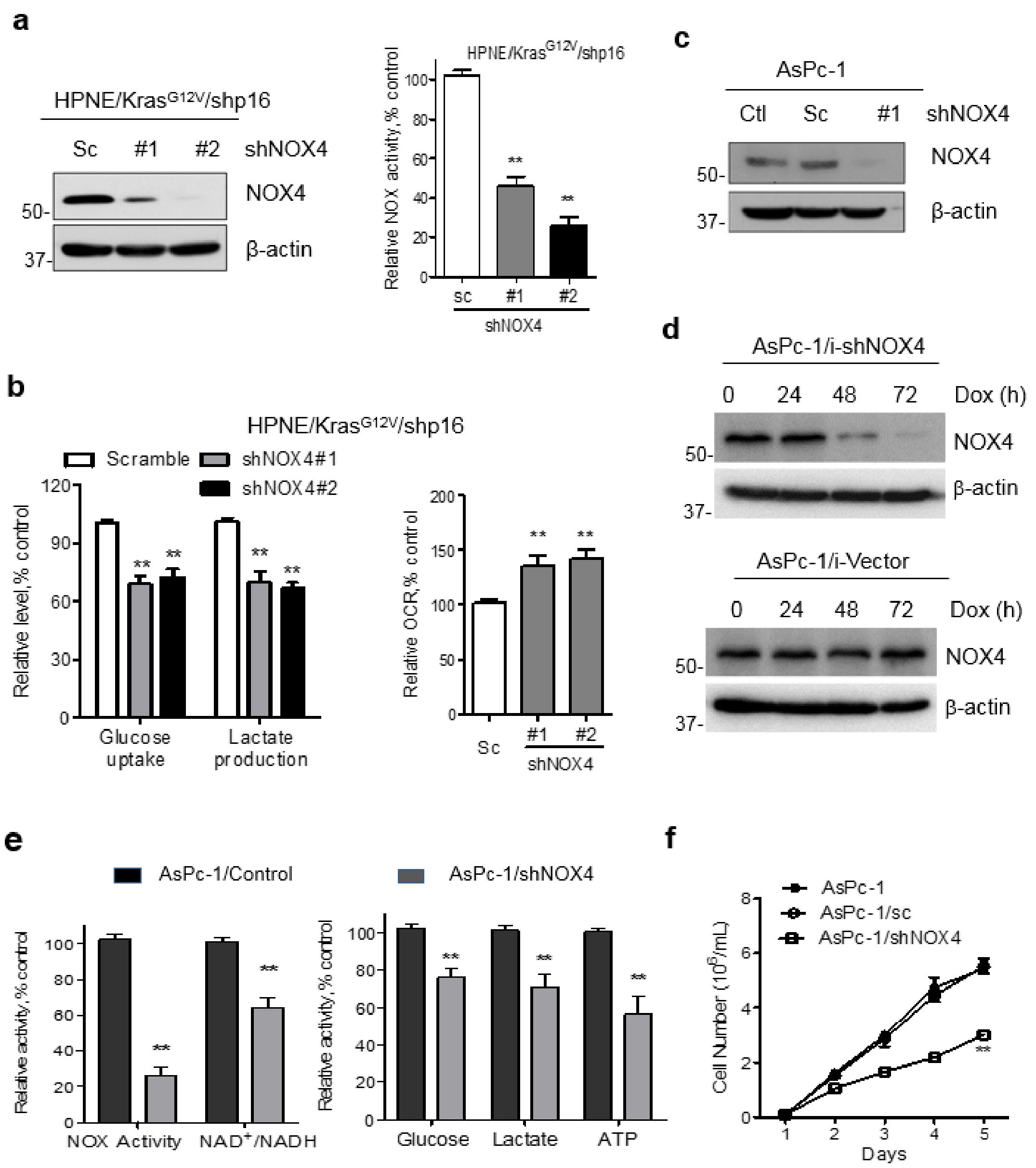
a
HPNE/Kras^G12V/shp16
Sc #1 #2 shNOX4
NOX4
β-actin
50-
37-
HPNE/Kras^G12V/shp16
Relative NOX activity, % control
sc #1 #2
shNOX4
c
AsPc-1
Ctl Sc #1 shNOX4
NOX4
β-actin
b
HPNE/Kras^G12V/shp16
Scramble
shNOX4#1
shNOX4#2
Relative level, % control
Glucose uptake
Lactate production
Relative OCR, % control
Sc #1 #2
shNOX4
d
AsPc-1/i-shNOX4
0 24 48 72 Dox (h)
NOX4
β-actin
AsPc-1/i-Vector
0 24 48 72 Dox (h)
NOX4
β-actin
e
AsPc-1/Control
AsPc-1/shNOX4
Relative activity, % control
NOX Activity
NAD+/NADH
Glucose
Lactate
ATP
f
AsPc-1
AsPc-1/sc
AsPc-1/shNOX4
Cell Number (10^6/mL)
Days

Supplementary Figure 5. Suppression of NOX4 leads to metabolic disruption and decreases pancreatic cancer cell growth. (a) The NOX4 knockdown efficiency and NOX activity was analyzed in NOX4-silenced HPNE/Kras^{G12V}/shp16 cells. (b) Glucose uptake and lactate production, and oxygen consumption rate were measured in primary NOX4-silend HPNE/Kras^{G12V}/shp16 cells derived from subcutaneous xenograft mice. (c, d) The expression of NOX4 was analyzed by immunoblotting in indicated cells. (e, f) NOX activity, NAD^+/NADH ratios and growth curve were determined in NOX4-silenced AsPc-1 cells. Each bar represents the mean ± s.d. (n=3). $^{**}P < 0.01$ for indicated comparison (Student unpaired *t*-test).

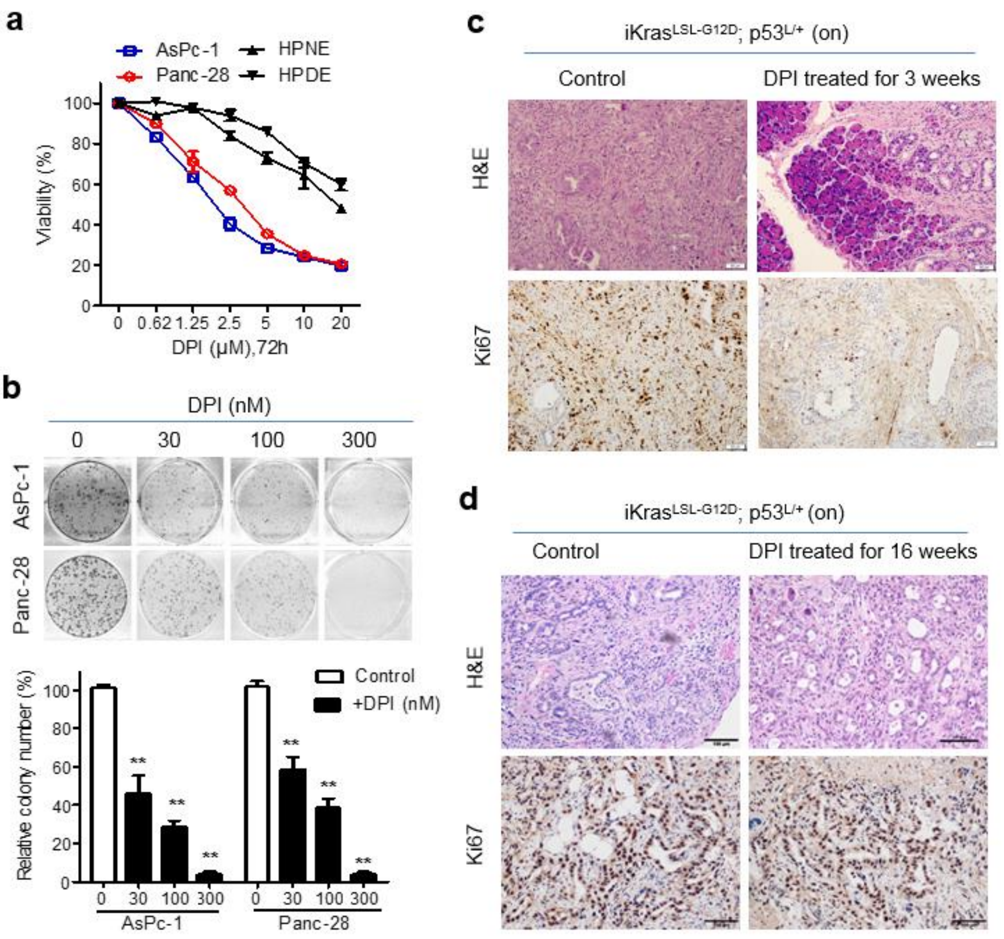

Supplementary Figure 6. DPI treatment for long time develops resistance in transgenetic *iKras; p53*$^{L/+}$ mice. (a) Cell viability was measured by MTS assay in indicated cells incubated with DPI for 72 h. (b) Images and quantification of the indicated cells incubated with DPI for 2 weeks were analyzed in a clonogenic assay. (c) Paraffin-embedded tumor sections from *iKras*$^{LSL\text{-}G12D}$*; p53*$^{L/+}$ mice, treated with or without DPI for indicated time, were stained with H&E or anti-Ki67 antibody (Scale bar: 100 µm). Each bar represents the mean ± s.d. (n=3). ***P* < 0.01 for indicated comparison (Student unpaired *t*-test).

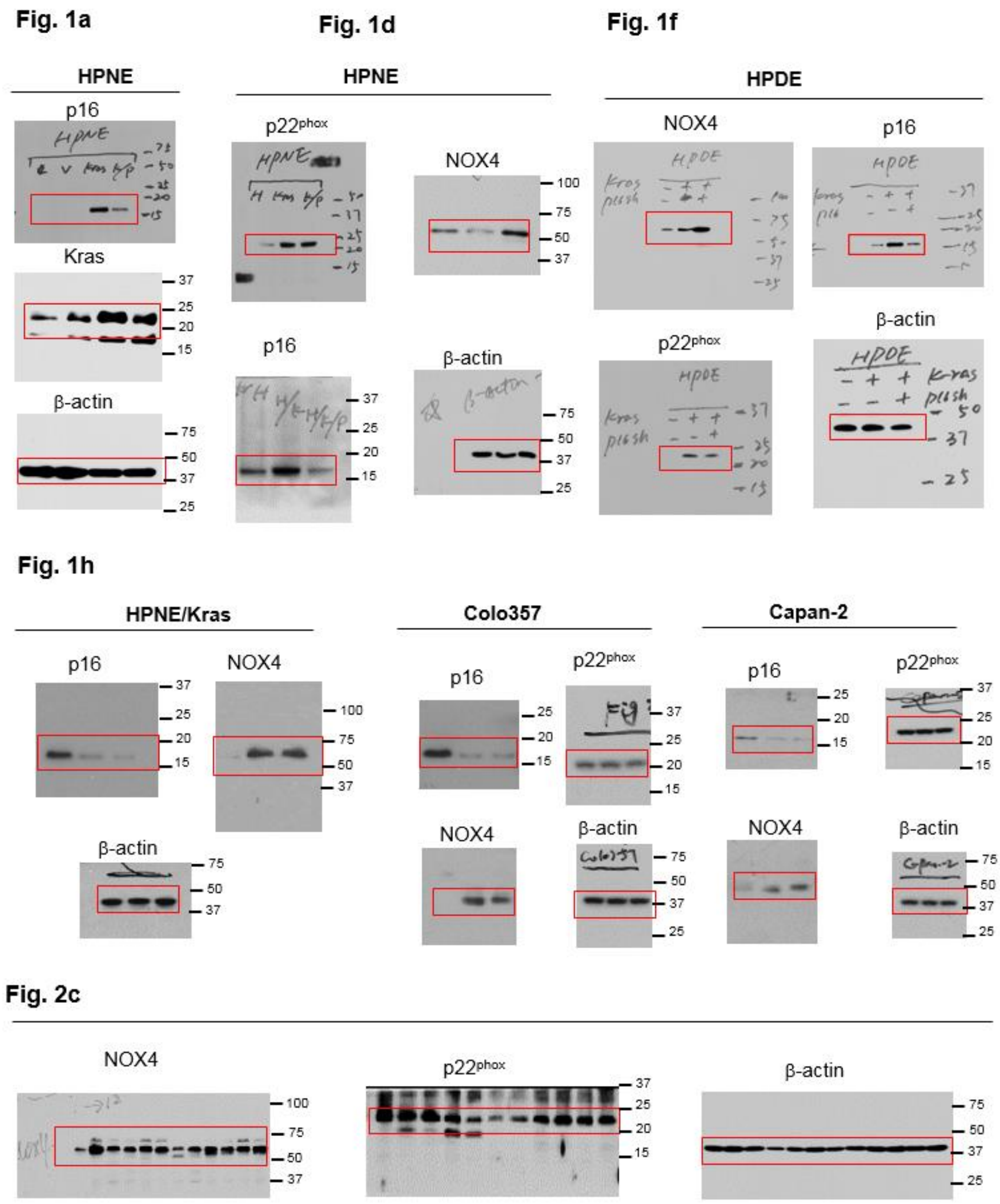

Supplementary Figure 7. Full scans of uncropped blots presented in the main paper. Red boxes indicate the cropped regions. Molecular weight markers are indicated in kDa.

Fig. 3c

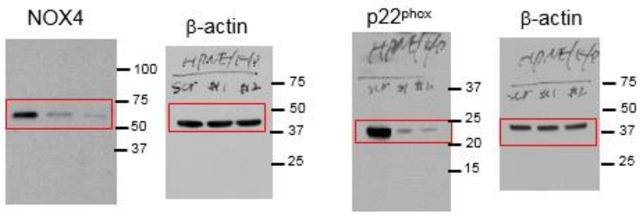

Fig. 3g

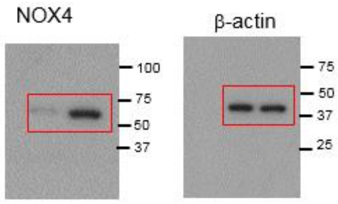

Fig. 4a

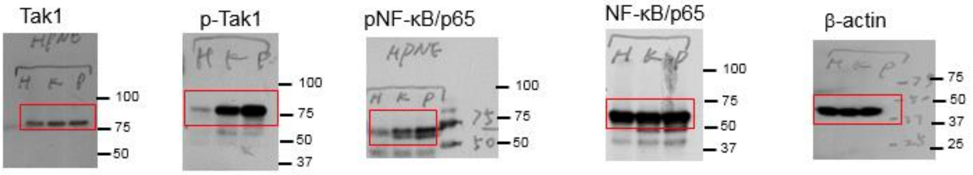

Fig. 4b

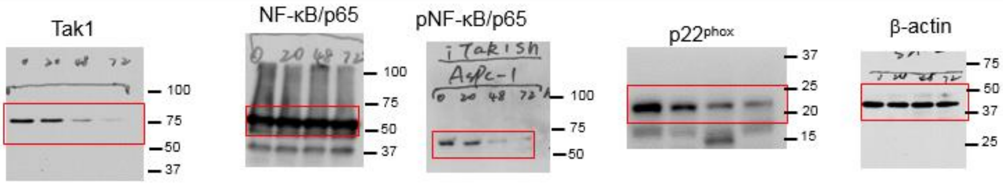

Fig. 4c

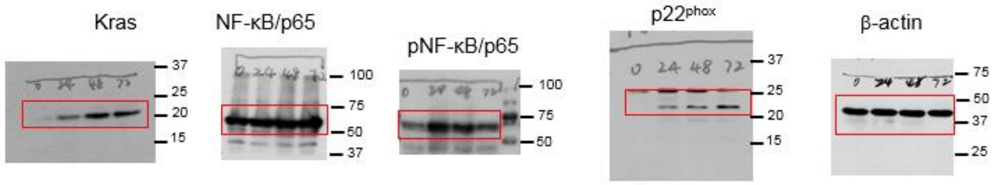

Supplementary Figure 7. Continued

Fig. 4e

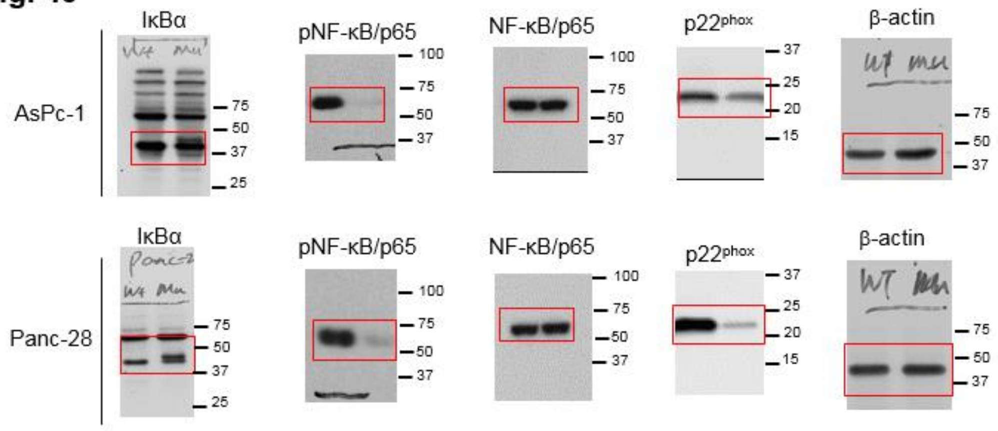

Fig. 4g

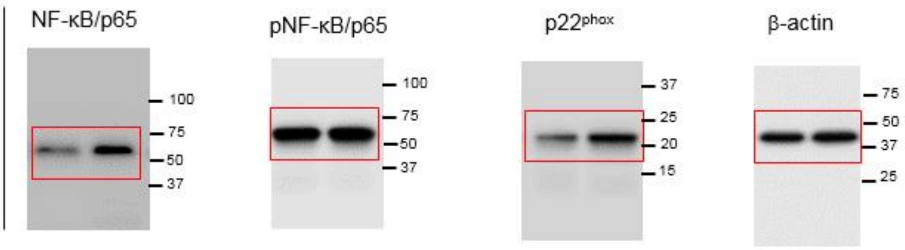

Fig. 5a

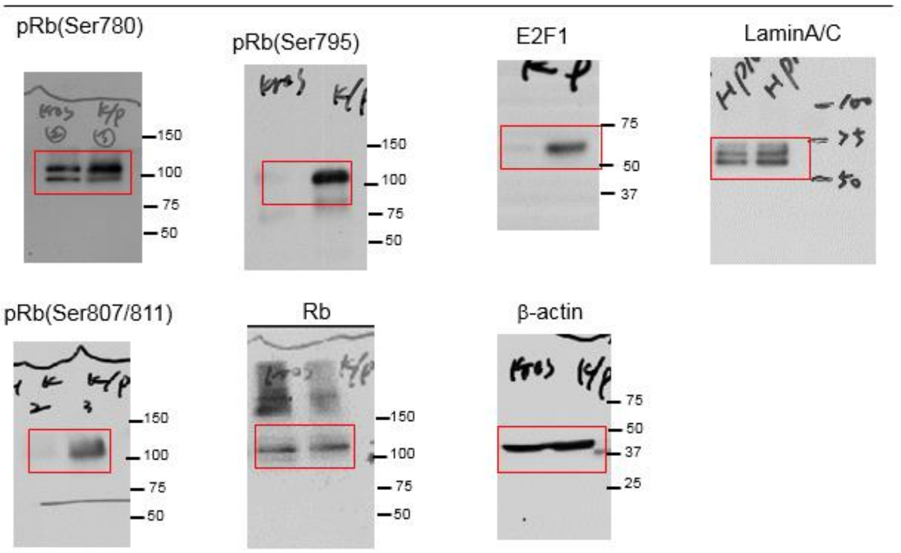

Supplementary Figure 7. Continued

Supplementary Table 1.

Highly increased genes (top 10) or decreased genes (top 10) in HPNE/Kras^{G12V}/shp16 cells compared with HPNE/Kras^{G12V} cells identified using cDNA microarray.

Rank	Gene name	Description	LogRatio
1	CRK	v-crk sarcoma virus CT10 oncogene homolog	2.46
2	FAM101B	family with sequence similarity 101, member B	2.37
3	**NOX4**	**NADPH oxidase 4**	2.31
4	MPDZ	multiple PDZ domain protein	2.25
5	IL13RA2	interleukin 13 receptor, alpha 2	2.25
6	BVES	blood vessel epicardial substance	2.13
7	ROBO1	roundabout, axon guidance receptor, homolog 1	2.13
8	TCF7L1	transcription factor 7-like 1	2.11
9	PGR	progesterone receptor	2.09
10	TRAM1L1	translocation associated membrane protein 1-like 1	2.05
1	NEFL	Neurofilament, light polypeptide	-3.11
2	SH3GL3	SH3-domain GRB2-like 3	-2.63
3	WWC1	WW and C2 domain containing 1	-2.52
4	ARMC4	Armadillo repeat containing 4	-2.50
5	EPDR1	Ependymin related protein 1 (zebrafish)	-2.46
6	PDZD2	PDZ domain containing 2	-2.46
7	VNN1	Vanin 1	-2.32
8	NFE2L3	Nuclear factor (erythroid-derived 2)-like 3	-2.29
9	FBN2	Fibrillin 2	-2.22
10	NHS	Nance-Horan syndrome	-2.15

Supplementary Table 2.

Quantitative PCR primer sequences of assessed genes.

Gene	Forward primer	Reverse primer	Length
Kras	5′-agagaggcctgctgaaaatg-3′	5′-agtctgcatggagcaggaaa-3′	703 bp
p16	5′-cccaacgcaccgaatagtta-3′	5′-accagcgtgtccaggaag-3′	173 bp
NOX1	5′-ttgtttggttagggctgaatgt-3′	5′-gccaatgttgacccaaggatttt-3′	106 bp
NOX2	5′-accgggtttatgatattccacct-3′	5′-gatttcgacagactggcaaga-3′	135 bp
NOX3	5′-cgtggcgcatttcttcaacc-3′	5′-gctctcgttaggggtgttgc-3′	106 bp
NOX4	5′-tgtgccgaacactcttggc-3′	5′-acatgcacgcctgagaaaata-3′	136 bp
NOX5	5′-ggctcaagtcctaccactgga-3′	5′-gaaccgtgtacccagccaat-3′	195 bp
$p22^{phox}$	5′-gtgtttgtgtgcctgctggagt-3′	5′-ctgggcggctgcttgatggt-3′	320 bp
NF-κB	5′-atgtggagatcattgagcagc-3′	5′-cctggtcctgtgtagccatt-3′	151 bp
E2F1	5′-acgctatgagacctcactgaa-3′	5′-tcctgggtcaacccctcaag-3′	249 bp
E2F2	5′-cgtccctgagttcccaacc-3′	5′-gcgaagtgtcataccgagtctt-3′	107 bp
E2F3	5′-agaaagcggtcatcagtacct-3′	5′-tggacttcgtagtgcagctct-3′	76 bp
E2F4	5′-caccaccaagttcgtgtccc-3′	5′-gcgtacagctagggtgtca-3′	85 bp
E2F5	5′-gggctgctcactaccaagttc-3′	5′-cctacacctttccactggatact-3′	188 bp
β-actin	5′-catgtacgttgctatccaggc-3′	5′-ctccttaatgtcacgcacgat-3′	250 bp
